# Best practice guidelines for stroke in Cameroon: An innovative and participatory knowledge translation project

**DOI:** 10.4102/ajod.v3i1.92

**Published:** 2014-11-21

**Authors:** Lynn Cockburn, Timothy N. Fanfon, Alexa Bramall, Eta M. Ngole, Pius Kuwoh, Emmanuel Anjonga, Brenda M.E. Difang, Shirin Kiani, Petra S. Muso, Navjyot Trivedi, Julius Sama, Sylvian Teboh

**Affiliations:** 1 Department of Occupational Science and Occupational Therapy, University of Toronto, Canada; 2 Cameroon Baptist Convention Health Board, Bamenda, Cameroon; 3 Undergraduate Medical Education MD Program, Faculty of Medicine, University of Toronto, Canada; 4 Buildings Bridges Cameroon (BBCAM), Yaoundé, Cameroon; 5 Director, Regional Hospital, Limbe South West Region, Cameroon; 6 Centre for Inclusion Studies, Bamenda, Cameroon; 7 Bamenda Regional Hospital, Bamenda, Cameroon; 8 Handicap International Federation, Erbil, Iraq; 9 St. Elizabeth’s Catholic General Hospital, Shisong, Banso, Cameroon; 10 School of Physiotherapy, RK University, Rajkot, India; 11 Ministry of Public Health, North West Region, Cameroon; 12 St. Joseph’s Children’s and Adults Home (SAJOCAH), Bafut, Cameroon

## Abstract

**Background:**

Although the adherence to stroke guidelines in high-income countries has been shown to be associated with improved patient outcomes, the research, development and implementation of rehabilitation related guidelines in African countries is lacking.

**Objectives:**

The purpose of this article is to describe how a group of front-line practitioners collaborated with academics and students to develop best practice guidelines (BPG) for the management and rehabilitation of stroke in adult patients in Cameroon.

**Method:**

A working group was established and adapted internationally recognised processes for the development of best practice guidelines. The group determined the scope of the guidelines, documented current practices, and critically appraised evidence to develop guidelines relevant to the Cameroon context.

**Results:**

The primary result of this project is best practice guidelines which provided an overview of the provision of stroke rehabilitation services in the region, and made 83 practice recommendations to improve these services. We also report on the successes and challenges encountered during the process, and the working group’s recommendations aimed at encouraging others to consider similar projects.

**Conclusion:**

This project demonstrated that there is interest and capacity for improving stroke rehabilitation practices and for stroke guideline development in Africa.

## Introduction

### Background

Stroke is a sudden loss of blood supply in the brain either due to a blockage (i.e. ischemic stroke) or rupture (i.e. haemorrhagic stroke) (Lindsay *et al*. 2010). It is an acute event that can have a sustained impact on many aspects of patients’ lives (including quality of life) and is associated with significant economic costs (Lundström *et al*. 2010; Norrving & Kissela 2013). In high-income countries, efforts have been made to address the risk factors of stroke and implement appropriate follow-up procedures to reduce stroke-related disability. However, a lack of attention to the prevention and treatment of stroke in low-income and middle-income countries (LMICs), including those in Africa, over the last four decades has caused the incidence of stroke to rise (Feigin *et al*. 2009). Whilst high-income countries have recorded a combined 42% reduction in stroke incidence, there has been an increase in stroke-related burden, morbidity and mortality in LMICs (Brainin, Teuschl & Kalra 2007; Feigin *et al*. 2009; Norrving & Kissela 2013). In African countries, increases in the population risk of stroke owing to hypertension, diabetes, lifestyle changes, demographic transitions and increased longevity, exist with challenges related to health infrastructure and treatment, such as under-detection and economic barriers to treatment (Balti *et al*. 2013; Brainin *et al*. 2007; Feigin *et al*. 2009; Ogun *et al*. 2005).

In under-resourced settings like Africa, six months poststroke mortality rates are as high as 44% – 47% (Garbusinki *et al*. 2005; Ogun *et al*. 2005). Many health and rehabilitation providers in Africa face difficult challenges when trying to provide the best quality services to their patients. Causes and challenges of these high rates include lack of human and material resources and poor infrastructure, such as inadequately equipped health facilities, electrical power and telecommunications systems as well as transportation systems (Garbusinki *et al*. 2005). In addition, opportunities for professional development are few, caseloads large and payment or incentives for the development of appropriate guidelines are low or non-existent.

The situation for stroke rehabilitation has been particularly neglected in practice and research in Africa (Kolapo & Vento 2011). However, outcomes can be improved and mortality rates reduced by introducing multidisciplinary stroke units and postdischarge community-based rehabilitation to sustain early gains in neurological function. Although poorly staffed health facilities and heavy caseloads are major reasons for decreased health care quality, a recent study carried out in rural Tanzania revealed that an increase in workforce alone was unlikely to improve quality of care (Maestad, Torsvik & Aakvik 2010). When both knowledge gaps and motivation of health care staff are addressed, performance can improve (Leonard & Masatu 2010).

One strategy successfully applied to improve the skills of health workers and quality of care globally is the use of best practice, practitioner-friendly, evidence-based guidelines (Baker *et al*. 2012; Manchikanti 2008; Qaseem *et al*. 2012; Reker *et al*. 2002; Woolf *et al*. 1999). Although the adherence to postacute stroke rehabilitation guidelines in high-income countries has been shown to be associated with improved patient outcomes (Duncan *et al*. 2005), incentives for the development and implementation of guidelines in low-income settings are few (Baker *et al*. 2012; Orem *et al*. 2012; Woolf *et al*. 1999) suggesting more attention should be paid to strategies to support guideline development and uptake, and changes in practice.

Many barriers to quality of care for stroke patients exist in Cameroon, a low-income African country. Stroke outcomes are poor due to insufficient qualified health staff, weak and inconsistent multidisciplinary team case management, the lack of stroke units, delays in initiating postacute stroke rehabilitation, and a significant shortage of rehabilitation staff and community-based rehabilitation services (Balti *et al*. 2013; Njamnshi *et al*. 2006). Rehabilitation services are offered in a small but growing number of health facilities in the North West Region (NWR) of Cameroon, one of the ten administrative regions of the country.

The purpose of this article is to describe a collaborative effort to develop best practice guidelines (BPG) for the management and rehabilitation of stroke in adult patients in the NWR. We present an overview of the process used to develop the guidelines, the successes and challenges encountered during their development, and end with recommendations and the identification of future work, aimed at encouraging others to consider similar projects in this and similar settings.

## Method

An eight-stage process was used by an interdisciplinary team to develop these best practice guidelines for stroke. The group was part of a larger initiative in which guidelines addressing other topics were also being developed. Therefore, this eight-stage process was determined in advance of beginning work on the guidelines as part of the larger project, and was based on internationally accepted recommendations on how to develop best practice guidelines (Atkins *et al*. 2004; Grimshaw, Eccles & Russell 1995; National Health and Medical Research Council [NHMRC] 1999; National Institute for Health and Clinical Excellence [NICE] 2007; New Zealand Guidelines Group [NZGG] 2001; Scottish Intercollegiate Guideline Network [SIGN] 2004). However, it was recognised that the process could be adapted by each team.

The eight stages were:

- identify and constitute working groups
- determine the scope and clinical questions
- documentation of current practices
- conduct systematic research for evidence
- critical appraisal the evidence
- draft guidelines and external review
- finalise the guidelines
- implementation and evaluation of the BPG.

Although the project is presented as a chronological process of eight stages, it is important to note that it was necessary to move backwards and forwards in the process or to revisit previously completed stages in light of new information, for example, returning to refine the scope statement or to update understandings of current local practices. The process described here took about four years; meetings were held 3–7 times per year.

### Step 1: Constitute the working group

When the project began, the first author called a meeting of experienced rehabilitation service providers and programme managers in the region who were identified as potential group members. There was a small pool of people to draw from – in the region at that time there were approximately four physiotherapists who were experienced in stroke rehabilitation, less than 10 community-based rehabilitation providers, and no occupational therapists or speech therapists. At the time there were no established health sciences university programmes or dedicated stroke units in the region. The group initially had four members. Working group members were included if they had educational and professional backgrounds in the areas of stroke rehabilitation, and their availability to commit to the project.

Two of the physiotherapists agreed to be co-leaders for the group. Doctors who expressed interest were also invited to join the group. Canadian volunteers (i.e. occupational therapists and a medical student) also participated in the project. Over the course of the project, the number of active working group members was between 6 and 10 members. The group included physical therapists, occupational therapists, physical therapy assistants, physicians, public health policy development and programme implementation specialists, researchers, and research students (see Table 1 for more details about the backgrounds of group members).

**TABLE 1:** Working group members’ professional background and domain of expertise.

Work group	Member	*N*	(n/N)
Service provider, private structure	TNF, SPM, ST	3	27.3
Service provider, public structure	EMN, BMED, PK	3	27.3
Health policy development and implementation	EMN, SK, JS	3	27.3
Monitoring of health policy implementation Domain of expertise	JS, PK	2	18.2
Occupational therapy	LC, SK	2	18.2
Physical therapy	TNF, BMED, SPM, ST, TN	5	45.5
Primary care	PK, EMN, LC, BMED, SPM, ST	6	54.5
Clinical stroke care	PK, EMN, TN	3	27.3
Community and public health and policy development	EMN, SK	2	18.2
Community development	EMN, LC, EA, SK	4	36.4
Advocacy	EMN, LC, TN, SK	4	36.4
Academic	LC, EMN, AB, TN	4	36.4
Community and public health policy implementation monitoring	JS, PK, SPM	3	27.3
Stroke and rehabilitation research	LC, EMN, AB	3	27.3
Members (partners) from high-income country partner institutions	LC, AB, SK	3	27.3
Health professional students involved over the course of the project	5	5	

TNF, Timothy N. Fanfon; SPM, Sister Petra Muso; ST, Sylvian Teboh; EMN, Eta M. Ngole; BMED, Brenda M.E. Difang; PK, Pius Kuwoh; SK, Shirin Kiani; JS, Julius Sama; LC, Lynn Cockburn; TN, Trivedi Navjyot; EA, Emmanuel Anjonga; AB, Alexa Bramall.

Within the group, encouragement and support from members and from the project leadership was instrumental in sustaining involvement and confidence in the process. Because none of the initial participants had direct experience in this type of guideline use or development, it took several meetings to develop an understanding of the expectations and projected outcome of the project. With time, members came to understand the goals of the project and stronger commitments to both the process and the outcome were made.

### Step 2: Determine the scope

In keeping with accepted practice in guideline development, the next step was for the working group to decide on what the guidelines would address, and what they would not address. The final scope statement is presented in Box 1.

BOX 1 **Scope statement.**

**Objective:**

To provide best practice recommendations related to rehabilitation for the acute stages of stroke in the North West Region of Cameroon.

In this document, the acute stages refer to the time that stroke occurs to approximately six months post-cerebrovascular accident (CVA). The scope of the guidelines includes practices related to assessment, referrals, management and rehabilitation.

This document focusses on initial assessment and initial treatment protocols in acute care facilities, the provision and monitoring of services, and discharge planning. Whilst these guidelines include references to medical management, detailed medical management is not the primary focus of these guidelines. Pharmaceutical treatment is important in stroke management but is outside of the scope of these guidelines and therefore is not included in these guidelines.

Rehabilitation settings that can use these guidelines: Inpatient hospitals, outpatient hospitals, and community-based rehabilitation programmes.

Disease(s) and/or condition(s): Stroke and Transient Ischemic Attack (TIA).

Age group(s): Individuals older than 16 years.

Intended users of the guidelines: Acute care management team and front-line staff involved in client care 0–6 months post-CVA; physical therapists, nurses, doctors, people who have experienced stroke and their family members and caregivers, community workers, other health workers if applicable.

The guidelines provide best practice recommendations related to rehabilitation for the early stages of stroke for adults in the *North-West Region* (NWR) of Cameroon. The scope of the guidelines includes practices related to assessment, referral, management, and rehabilitation from the time the stroke occurs to approximately six months after. Both ischemic and haemorrhagic stroke are included. The group decided to focus on adults, as the needs of children can be quite different from adults, and felt that a separate guideline should be developed for children.

At first, the guidelines scope was limited to stroke rehabilitation, mainly because the initial intention was the development of guidelines for the improvement of stroke rehabilitation outcomes. As time went on, and based on clinical experiences expressed by working group members on patient needs, the group decided to include some aspects of assessment and management. It became clear during group discussions that in this practice context diagnosis and management of acute stroke were closely linked with early rehabilitation practices, not distinct. There were no other stroke guidelines for this setting. However, this decision presented another dilemma: the group recognised that whilst there was a need to fill the existing gaps in guidelines for diverse aspects of stroke care in Cameroon in general, and in the NWR in particular, not all aspects could be handled in these guidelines given the resources available. Group members revisited and debated the scope of the guidelines several times, always keeping in mind the goal of having a real impact on meeting the needs of patients and also considering the available resources. Although acute often means within the first month poststroke (Lindsay *et al*. 2010), the group discussed this terminology and felt that in this context ‘acute’ was interpreted to mean from onset up to six months.

### Step 3: Documentation of current practices

In order to create guidelines appropriate to a local context, current practices need to be understood and documented. To do so, group members shared their familiarity with current practices, talked with colleagues, and reflected on what they had learned from clients and the family members of stroke patients. In 2009, when we started to collect information about the services provided for stroke patients, there did not appear to be a systematic referral system for stroke patients or any dedicated stroke units in the region.

Systematic searches were conducted for literature about stroke rehabilitation in the NWR, and in Cameroon generally, using the University of Toronto databases and Google Scholar. No research articles were identified.

The results of these discussions were written up in the guidelines document under ‘Current Situation and Current Practices in the North West Region’ to guide the next steps of the process; in 2012, this section was updated.

As of 2012, there were 19 health districts with 337 health units in the NWR. These included hospitals, medicalised health centres (i.e. those that have a doctor) and health centres without doctors, many of which were in rural and remote areas. Although none of the health units were dedicated to stroke care or had a stroke unit, and just a handful provided specific stroke rehabilitation programmes, all could receive people experiencing a stroke. Of these health units, we were aware of six institutions that provided stroke rehabilitation services. Each of these six institutions had at least one physiotherapist and physiotherapy assistants.

### Step 4: Search for evidence, revising the process, and deciding to adapt the Appraisal of Guidelines for Research and Evaluation (AGREE) process

Once there was a clearer understanding of what the current situation was, the group identified key questions and areas of focus, and embarked on a systematic literature search using electronic databases (Medline, PubMed and Google Scholar) to learn more about possible answers. Because the body of literature related to stroke is extremely large, the search was focussed on identifying research evidence especially from Cameroon and Africa, with relevant articles from Europe and North America as needed.

The search terms used included the terms stroke, cerebral vascular accident, cerebrovascular accident (CVA), Cameroon and Africa. From identified articles two additional strategies were used: reference lists of retrieved articles were also read, and the names of key authors were also used as search terms. Evidence from articles primarily addressing stroke from Cameroon and other Africa countries was reviewed to identify any key information or recommendations that could contribute to formulation of the guidelines, and to assist the working group to become more familiar with this body of research. This resulted in a list of 32 articles that had direct relevance to stroke rehabilitation in Cameroon.

The guideline development process initially used by the group began with the assumption that the working group would collect and review evidence to develop the practice guidelines. However, it became clear that many guidelines related to stroke had already been developed, and so it was decided to use the AGREE process, a well-established method to select and adapt publicly available guidelines (AGREE Collaboration 2003) and to base our guidelines on these.

The process for selecting guidelines is included as an appendix in the stroke guidelines document, including the scoring process used. Our priority was to look for guidelines from LMIC (low-income and middle-income countries) to reflect as closely as possible the context of Cameroon, and which could be accessed online. Guidelines from Africa would be most relevant, followed by guidelines from other LMIC, such as in parts of Asia and South America. We were able to identify one African stroke guideline from South Africa (Bryer *et al*. 2011). Although there are guidelines from China and other Asian regions, these are not available to the public. We were able to access guidelines from the Philippines as well as from Singapore (Singapore Ministry of Health 2009).

A second priority was to incorporate a document not from a LMIC but as an example of a recent guideline of high quality. The Stroke Canada guidelines (Lindsay *et al*. 2010) were recent at the time, and are a synopsis of existing guidelines from the American Academy of Neurology, other Canadian and American organisations, Chinese stroke trials, and guidelines from Australia, New Zealand, and the Scottish Intercollegiate Guidelines Network. We therefore chose this guideline because it covered a very broad range of guidelines from around the world and incorporated important points from multiple sources and different international perspectives. The authors of this guideline also followed the AGREE process for drafting the full Canadian stroke guidelines.

Through this process we identified four published guidelines to adapt Canada, Philippines, Singapore and South Africa and several research articles to review, for the guidelines document which we were preparing.

### Step 5: Appraising the evidence

Recommendations applicable to the local context were selected and progressively adapted. These recommendations came from both the four identified guidelines documents identified in Step 4 and the research articles.

Recommendations were graded as A, B, C and D, adapted from the four levels of evidence, used by the Scottish Intercollegiate Guidelines Network (SIGN 2004). Level A evidence was the strongest, with recommendations derived from randomised control trials. Levels B, C, and D were used to indicate how strong the evidence was based on other published work, expert opinion, and formal consensus of local experts and stakeholders, respectively.

Each draft recommendation from the four source guidelines was discussed in detail by the members of the group with respect to evidence, and for applicability and adaptation to the local context until consensus was reached. This process took place over the course of several face-to-face meetings and through the use of written comments on draft documents. In some instances the recommendation was agreed on as stated, for others, it was revised to be more relevant. There was significant discussion about the recommended health facility level for each guideline. If the group felt that the original intent of a recommendation could not be maintained, the recommendation was removed.

Having four guideline documents was also useful for deciding how to present the final document. By comparing the four documents, the organisational structure for the guidelines document was also considered. The guidelines were reformatted until members felt that they were reflective of the health system in the region.

At the end of Step 5, a draft document was developed for external review. This document had the main categories seen in the final document.

### Step 6: Draft guidelines external review

Seventeen experts, with local and international experience, were approached to critically review the guidelines, emphasising the applicability to the local context (see Table 2 for their locations and professional backgrounds). Reviewers were identified by their publications or because they were known to the members of the working group. Suggestions made by the reviewers were discussed and changes made; the final guidelines document was the outcome of Step 6 and is available at http://icdr.utoronto.ca

**TABLE 2:** Reviewer characteristics.

Primary work location	Location	Professional background	Work group member domain of expertise
Mbingo	Boyo Division	Medical doctor	Clinical stroke care
Hospital	Bui Division	Medical doctor	Clinical stroke care; community and public health
Special programme at a hospital	Bui Division	Research assistant; nurse	Clinical stroke care
Private training school for health personnel in Kumbo	Bui Division	Principal; nurse	Academic; clinical stroke care
Hospital	Bui Division	Doctor	Clinical stroke care
Hospital, in Bafut	Mezam Division	Medical doctor	Clinical stroke care
Rehabilitation centre	Mezam Division	Physiotherapist	Clinical stroke care
Bamenda	Mezam/Region	Medical doctor; administrator	Clinical stroke care; community and public health; health policy
Regional hospital, in Bamenda	Mezam/Region	Medical doctor	Clinical stroke care; community and public health; health policy
Yaounde	National	Doctor or researcher	Neurology; academic; research
Bamenda	North West Region	Public health	Community and public health
Bamenda	North West Region	Programme administration	Community and public health administration
Bamenda	North West Region	Medical doctor	Clinical stroke care
Mbingo/Banso	North West Region	CBR Programme	Community-based stroke care
Mbingo	North West Region	CBR Programme	Community-based stroke care
Training school	North West Region	Nursing; Director of a Nursing School	Academic; clinical stroke care
Vancouver, Canada	Vancouver	Physical therapist	Clinical stroke care

### Step 7 and Step 8: Guidelines, distribution and implementation

The final stages of the process are to distribute, implement, and facilitate the use of the best practice guidelines, and this has been underway since 2013. To make improvements in stroke services within the region, the guidelines are being distributed to the Ministry of Public Health, hospitals, and institutions providing stroke rehabilitation services, general practitioners, community-based rehabilitation programmes, educational programmes, and other health related programmes in the region. Currently, funding is not available for a large-scale dissemination effort or to systematically monitor the uptake.

The guidelines are available in hard copy and in soft copy for those who have computer access (many front-line providers have no or very limited computer access). Funds are being sought to support the Ministry of Public Health and service providers to carry out training workshops for users and to assess the ease of use and impact. The development of a more user friendly format, such as application for mobile devices, is a goal for future work. We are exploring ways to develop learning communities of practitioners, because these kinds of opportunities were identified by the members as important.

## Results

### Outcome and recommendations

As indicated above, the key outcome of the project is a document titled ‘Best Practice Guidelines for the Management and Rehabilitation of Stroke in the North West Region of Cameroon’. This document provides an overview of the current health infrastructure, locations of organisations and descriptions of human resources providing stroke rehabilitation services, and approximate costs of consultation, admission, and physiotherapy sessions. In total, there are 83 practice recommendations plus recommendations related to implementation of the guidelines.

As indicated in Table 3, the recommendations are applicable for three types of practice situations and service provision, namely:

- inpatient rehabilitation
- outpatient rehabilitation
- community-based rehabilitation (CBR).

There are three sets of additional recommendations specifically for areas identified by experts in the region as priority – dysphagia, depression, and shoulder pain – applicable in all types of settings.

Annexed to the guidelines are practice oriented tools including a Cameroon Stroke Screening (CSS) tool developed for use in the NWR based on well-known scales (Adams *et al*. 2007; Crocco *et al*. 2007; Hurwitz *et al*. 2005; Kothari *et al*. 1999; Nau *et al*. 2010; Werner 1992), an example of a referral form, a screening tool for depression, and a guide to assessing nutritional status and dysphagia.

Key outcomes of the project include increased capacity of team members to understand and translate research to practice, significantly improved networking and collaboration amongst stroke rehabilitation providers in the region, and increased interest for adapting the guidelines to other regions and for holding conferences related to stroke and rehabilitation.

## Discussion

This project began with the goal of following accepted practices for the development of best practice guidelines, whilst adapting the process to an African, low-resourced context. Although the process took longer than initially planned, a final guideline document has been produced and is being implemented. Despite the many difficulties encountered on the way, the project has provided both tangible and intangible benefits to practitioners, and ultimately to clients.

Recognising that this was a first experience in evidence-based practice and guideline development for most team members, at the end of the project, the group identifies the importance of taking time to engage in a collective process of reflection. In the first year, many of the managers in the organisations involved did not understand practice guidelines, or the concepts of evidence-based or best practices; now, five years later, team members are encouraged that they have a tangible outcome to share with managers, administers and policy makers, and hope that other opportunities will arise to develop and use guidelines or similar tools in their work.

In terms of the implementation of the guidelines, both informal and formal implementation plans and experiences have resulted from this project. Informal implementation was evident before the guidelines were completed: changes in practices, such as increased emphasis on team meetings and referrals for rehabilitation, were noticed by the end of the second year, and these changes continued as the project carried on. Whilst the literature on guideline development presents a linear process, with guideline implementation following the development process, our experience has been closer to what has been suggested by Kolapo and Vento (2011:710): changes in practice should be implemented as soon as possible because improvements in stroke care are urgently needed in Africa. Because these practice changes were not fully anticipated at the beginning of the project, and because the funding for the project did not extend to implementation, we did not have a built-in mechanism for tracking these changes. However, in the process of finalising the guidelines in Years 3 and 4, participants reflected on the changes that they had seen in their own learning and practices, and many of these perspectives are included in this article.

Overall, the participatory approach brought together experienced professionals, researchers, and students, with diverse perspectives and opinions and helped to create a document representing these perspectives and opinions. Participants enjoyed the opportunity to learn about best practice guidelines as a systematic approach to dealing with pertinent health care issues. Health care infrastructure, human resource issues, and cost of services in the NWR were clarified throughout the guideline development process. Having these discussions allowed group members to consider how these systems could be improved, including describing the roles and responsibilities of care providers at each level. Some members of the project directly involved in patient care have already begun to implement some of the recommendations in their day to day practice.

One of the broader and more fundamental goals of this collaborative work was to support the retention and morale of health and rehabilitation workers in the region. Whilst this outcome has not yet been formally evaluated, members of the group talked about improved job satisfaction, pride and confidence in their clinical decision-making in having improved status and leadership in their workplaces, and in having other professional development opportunities arise that they can take advantage of because of their experience in this project. These opportunities included networking with colleagues, presenting at professional conferences, participating in international meetings, and sharing their learning through peer-reviewed articles such as this one.

### Challenges and limitations

Although there was enthusiasm about the project initially, some participants did not have the time to participate as it was not seen as part of their paid work; most team members volunteered their unpaid time to the project. It took group members several years to comprehend the methodology of the guideline development process, to work through each stage, and to fully understand the expectations at each stage of the process. Motivation waned at times; support and encouragement from other group members was critical to sustaining involvement. Enthusiasm and participation increased as members saw the guidelines take shape, and when they received positive feedback from colleagues on draft versions.

Consensus building around a recommendation required considerable discussion, especially when there were diverging perspectives. Meetings typically lasted between three and six hours, and were often held on weekends to accommodate busy schedules. It was difficult for busy people to devote time to attend meetings, but many sacrificed their time and effort repeatedly, and found that they enjoyed the spirited discussions. One of the significant benefits of the project was the opportunity for members to learn from each other, to share ideas about best practices, and to learn about what was going on in neighbouring organisations and cities. The outcomes are better because there was adequate time to discuss issues in detail.

Group members were consistently concerned about the applicability of the guidelines to the NWR. A challenge was to come out with a document that was applicable for the region, and which could possibly be applied in other regions of the country. At times the group found itself going off track to discuss and share information about the current situation in the region or about what was happening in other places; this was seen to a crucial benefit of meeting together. Whilst there are substantial gaps between what is recommended in guidelines from wealthy countries compared to what is available to the NWR, having these discussions also assisted members to consider goals and what might be possible in the future for stroke rehabilitation. The group had to combine their professional experiences to adapt the recommendations to the local realities, rather than solely relying on evidence from existing guidelines. Given the limitations and restrictions in the health system (Echouffo-Tcheugui & Kengne 2011; Tantchou Tchoumi & Butera 2013) and the fact that there is no ongoing, dedicated budget for dissemination and implementation, group members were also challenged to consider creative and low cost ways of implementing the guidelines.

## Recommendations

The group makes the following recommendations for others when considering similar initiatives:

1. **Ensure that there is a leadership team committed to the development of locally relevant guidelines and of the inclusion of a wide range members**: It is important to take the time to educate all people involved in the project, including key managers and care providers in relevant organisations and policy makers. For example, at times, attempts to involve managers in the organisations involved took longer than expected, and was perceived to be slowing down the project.
2. **It is important to create communities of practitioners who are interested in improving health care services:** This project was part of a larger initiative learning about best practices and evidence-based practices in rehabilitation in the region, and therefore several people and groups working on different topics came together periodically to share experiences and tools. These broader perspectives helped members to see the benefits of their own efforts and to more easily grasp the processes involved in the development of guidelines.
3. **Ensure that expectations are discussed and that enough time for extended discussions is allotted within meetings.**
4. **Recognise that many types of expertise and skill are useful. Use the experience of volunteer professionals and students to assist with components of the project**: The project relied heavily on the many contributions of expert health professional volunteers and students, and could not have been completed without their work. Volunteers and students were able to organise meetings, search, and review the literature, participate in evaluation, and contribute substantially to group discussions.
5. **Allow for sufficient financial resources:** This kind of project can be quite costly due to coordination, transportation, and meeting costs, even when leaders and participants are not paid. This was the first time that this team had carried out such a project, so although careful budget forecasting was done at the beginning, the initial funding was not sufficient to carry out the full objectives of the project.

### Future work

This project has identified further needs and provided inspiration for new projects such as the following:

- Need for more educational opportunities about better practices, guidelines, service pathways, and implementation strategies specific to this region and the Cameroon context in general: Education and professional development is needed due to severe limitations in resources and the very limited opportunities health professionals have to keep up with changes in practice. More attention should be given in local health professional educational programmes to evidence-based and best practices. Having succinct and accessible learning tools could have a significant impact on patient outcomes.
- Lack of research on stroke locally: The group applauds those researchers who are contributing to this body of knowledge, and encourage innovative ways of including front-line professionals, health system managers, and students to develop the knowledge base further.
- Significant need for research and evaluation on how practice guidelines can be developed, adapted, and implemented for low-income countries: These include a need for more appropriate alternatives to the adaption process recommended by collaborations which are well resourced. For example, there is a need for the development and evaluation of colourful, pocket-sized and soft format (cellular phone) applications that are appropriate to the resources available in this local context.
- The need for ongoing implementation and evaluation of these kinds of projects is also evident.
- The team was impressed with the commitment shown by the front-line practitioners in this project; projects such as this one should be encouraged to continue by managers and funders: The group willingly shared their reflections, improved understandings and frustrations of how guidelines can be adapted, developed, and used in low-income contexts.

## Conclusion

This project produced a best practice guideline document on stroke care and rehabilitation in a low-income country. The guidelines have the potential to improve service provision and patient outcomes in the NWR, and in other parts, of Cameroon. Although it was not possible to formally study its implementation due to the limited resources available to the project team, there is some evidence from this experience that the process of developing guidelines can lead to improved practices and can influence programme managers; this is an area that deserves further exploration and research.

The people who were members of this team have a new appreciation for what is required to improve practice: the ability to critique and use evidence, a commitment to dealing with the difficulties which will inevitably arise in a low-resourced context, skills in team facilitation and dedicated collaboration across organisations and disciplines, and the need for sufficient resources to bring people together in various ways. The development of these guidelines by a group of local practitioners, in the context of the larger project, has demonstrated that there is interest and capacity for improved practices in resource-limited contexts which should continue to be supported.
